# Errors in Table and Supplement 1

**DOI:** 10.1001/jamanetworkopen.2023.41784

**Published:** 2023-10-19

In the Consensus Statement titled “Guidelines for Diagnosis and Management of Infective Endocarditis in Adults: A WikiGuidelines Group Consensus Statement,”^1^ published July 31, 2023, there were errors in Table 2 and eTable 3 in Supplement 1. In Table 2 and eTable 3 in Supplement 1, the units for penicillin should have been micrograms per milliliter. This article has been corrected.^1^

## References

[1] McDonald EG, Aggrey G, Tarik Aslan A, . Guidelines for diagnosis and management of infective endocarditis in adults: a WikiGuidelines Group consensus statement. JAMA Netw Open. 2023;6(7):e2326366. doi:10.1001/jamanetworkopen.2023.26366 37523190

